# Standardization and optimization of the hiPSC-based PluriLum assay for detection of embryonic and developmental toxicants

**DOI:** 10.1007/s00204-024-03870-8

**Published:** 2024-10-04

**Authors:** Andreas Frederik Treschow, Anne Marie Vinggaard, Maria João Valente

**Affiliations:** Cell Toxicology Team, National Food Institute, Technical University of Denmark, Kemitorvet B204, 2800 Kgs Lyngby, Denmark

**Keywords:** Embryotoxicity, hiPSCs, PluriLum, Cardiomyocyte, Differentiation, NAMs

## Abstract

New approach methodologies (NAMs) for predicting embryotoxicity and developmental toxicity are urgently needed for generating human relevant data, while reducing turnover time and costs, and alleviating ethical concerns related to the use of animal models. We have previously developed the PluriLum assay, a *NKX2.5*-reporter gene 3D model using human-induced pluripotent stem cells (hiPSCs) that are genetically modified to enable the assessment of adverse effects of chemicals on the early-stage embryo. Aiming at improving the predictive value of the PluriLum assay for future screening purposes, we sought to introduce standardization steps to the protocol, improving the overall robustness of the PluriLum assay, as well as a shortening of the assay protocol. First, we showed that the initial size of embryoid bodies (EBs) is crucial for a proper differentiation into cardiomyocytes and overall reproducibility of the assay. When the starting diameter of the EBs exceeds 500 µm, robust differentiation can be anticipated. In terms of reproducibility, exposure to the fungicide epoxiconazole at smaller initial diameters resulted in a larger variation of the derived data, compared to more reliable concentration–response curves obtained using spheroids with larger initial diameters. We further investigated the ideal length of the differentiation protocol, resulting in a shortening of the PluriLum assay by 24 h to 7 days. Following exposure to the teratogens all-*trans* and 13-*cis* retinoic acid, both cardiomyocyte contraction and measurement of *NKX2.5-*derived luminescence were recorded with a similar or increased sensitivity after 6 days of differentiation when compared to the original 7 days. Finally, we have introduced an efficient step for enzymatic dissociation of the EBs at assay termination. This allows for an even splitting of the individual EBs and testing of additional endpoints other than the *NKX2.5*-luciferase reporter, which was demonstrated in this work by the simultaneous assessment of ATP levels. In conclusion, we have introduced standardizations and streamlined the PluriLum assay protocol to improve its suitability as a NAM for screening of a large number of chemicals for developmental toxicity testing.

## Introduction

To assess the safety of new drugs and chemicals, companies are required to test for and assess potential toxic effects on the developing fetus (Luconi et al. 2022; Piersma et al. 2022). The framework for the early embryonic toxicological assessments is outlined in the OECD test guideline 414 (OECD 2018) and ICH harmonized guideline S5 (European Medicines Agency 2020), which entail exposing two species of mammals to the chemical or drug in question throughout a full gestation period, and assessing the effects on the litter. This approach is problematic for several reasons. One of the most notable disadvantages of this approach is the interspecies differences inherent to the extrapolation of data from experiments conducted on predominately rats and rabbits for the assessment of toxicity in humans (Burton et al. 2016; Braakhuis et al. 2019; Schmidt et al. 2021). In addition, there are obvious downsides to the use of animal models related to ethical concerns, their scalability and costs, as well as how time consuming they can be (Rovida and Hartung 2009).

There is an urgent need for the development of screening tools and test systems that can provide human relevant developmental toxicity data in a less expensive and faster manner. The development of new approach methodologies (NAMs) based on in vitro test systems containing human stem cells has been shown to have the potential to fill this gap (Moradi et al. 2019; Piersma et al. 2022; Schmeisser et al. 2023; Kaplan et al. 2024).

Advancements in cell culture techniques, particularly the transition from conventional 2D cultures to more physiologically relevant 3D structures, further enhance the fidelity of in vitro models by more closely resembling in vivo conditions (Fleischer et al. 2019; Zuppinger 2019; Groen et al. 2024).

Our team has previously developed the PluriLum reporter gene assay (Lauschke et al. 2021b), capitalizing on the formation of embryoid bodies (EBs) combined with directed cardiac differentiation—a process mirroring processes present during early embryonic development (Brickman and Serup 2017; Zeevaert et al. 2020). We have previously shown that the PluriLum assay can detect well-known embryotoxic substances such as thalidomide, 5-fluorouracil, and epoxiconazole, whereas non-embryotoxic compounds such as ascorbic acid and folic acid were tested negative (Lauschke et al. 2021b; Treschow et al. 2024).

Although this assay has been demonstrated to have a reasonable predictive potential, some issues regarding the robustness of the assay were noted along past experiments, including random failure of differentiation leading to resection of full experiments, and large deviation of data resulting in loss of sensitivity. To address these issues, and to enhance the performance of the PluriLum assay, we have in this work undertaken the task of optimizing the experimental protocol by: (1) introducing quality standards at the beginning of the protocol, aiming at increasing the overall reproducibility of the assay; (2) decreasing the duration of the protocol while maintaining the sensitivity of the assay; and (3) including a step of dissociation of the EBs at the end of the protocol and thorough splitting of cells for testing of different endpoints, to maximize the utilization of the assay.

## Materials and methods

### Reagents and chemicals

All reagents for the PluriLum differentiation protocol were identical to those described in Treschow et al. (2024) except for the stem cell growth medium that was changed from mTeSR™1 to an improved formulation, mTeSR™ Plus, obtained from STEMCELL Technologies Inc. (Vancouver, Canada). Corning^®^ Matrigel^®^ hESC-Qualified Matrix and Corning^®^ ITS Premix Universal Culture Supplement were obtained from Corning Inc (NY, USA). TrypLE™, Penicillin–Streptomycin-Glutamine (PSG), KnockOut™ DMEM medium, human fibroblast growth factor-basic (FGF2), activin A, 60 mm cell culture dishes, and 96-well Polystyrene Conical Bottom MicroWell™ plates were supplied by Thermo Fisher Scientific Inc. (Massachusetts, USA). L-Ascorbic acid 2-phosphate trisodium salt (Asc), sodium selenite, human transferrin, all-*trans* (a*t*RA; CAS number 302-79-4), and 13-*cis* retinoic acid (13-*cis*RA; CAS number 4759-48-2) were purchased from Merck KGaA (Darmstadt, Germany). Rho kinase inhibitor was obtained from Abcam Plc (Cambridge, UK). 6-(2-(4-(2,4-Dichlorophenyl)-5-(4-methyl-1H-imidazol-2-yl)-pyrimidin-2-ylamino)ethyl-amino)-nicotinonitrile (CHIR99021) was purchased from Axon Medchem (Groningen, the Netherlands). 4-(2-Methyl-4-pyridinyl)-N-[4-(3-pyridinyl)phenyl]benzeneacetamide (Wnt-C59) and human bone morphogenetic protein 4 (BMP4), and were procured from Bio-Techne (Minnesota, USA).

### Cell culture maintenance

The cell line used for the PluriLum assay is the BIONi010-C-*NKX2.5*-T2A-*Nluc*-44.37, which was established and quality assessed in collaboration with Bioneer A/S (Hørsholm, Denmark) (Lauschke et al. 2021b). The hiPSCs culture was maintained on Matrigel^®^-coated cell culture dishes in mTeSR™ Plus medium. Cells were kept at 37 °C and 5% CO_2_ in a humid environment. Culture medium was changed every day, or second day and cultures were split approximately once a week using 0.02% EDTA in DPBS.

### Cardiomyocyte differentiation

The hiPSCs were differentiated into cardiomyocytes as previously described (Lauschke et al. 2020). Near confluent hiPSC cultures were dissociated into single cells by incubation with TrypLE™ for 4 min at 37 °C. Cells were then resuspended in medium and single cells were then seeded into 96-well Polystyrene Conical Bottom MicroWell™ plates in accordance with the desired density in 100 µl medium/well and spun down at 500*g* for 5 min (Day-1, D-1).

**Fig. 1:**
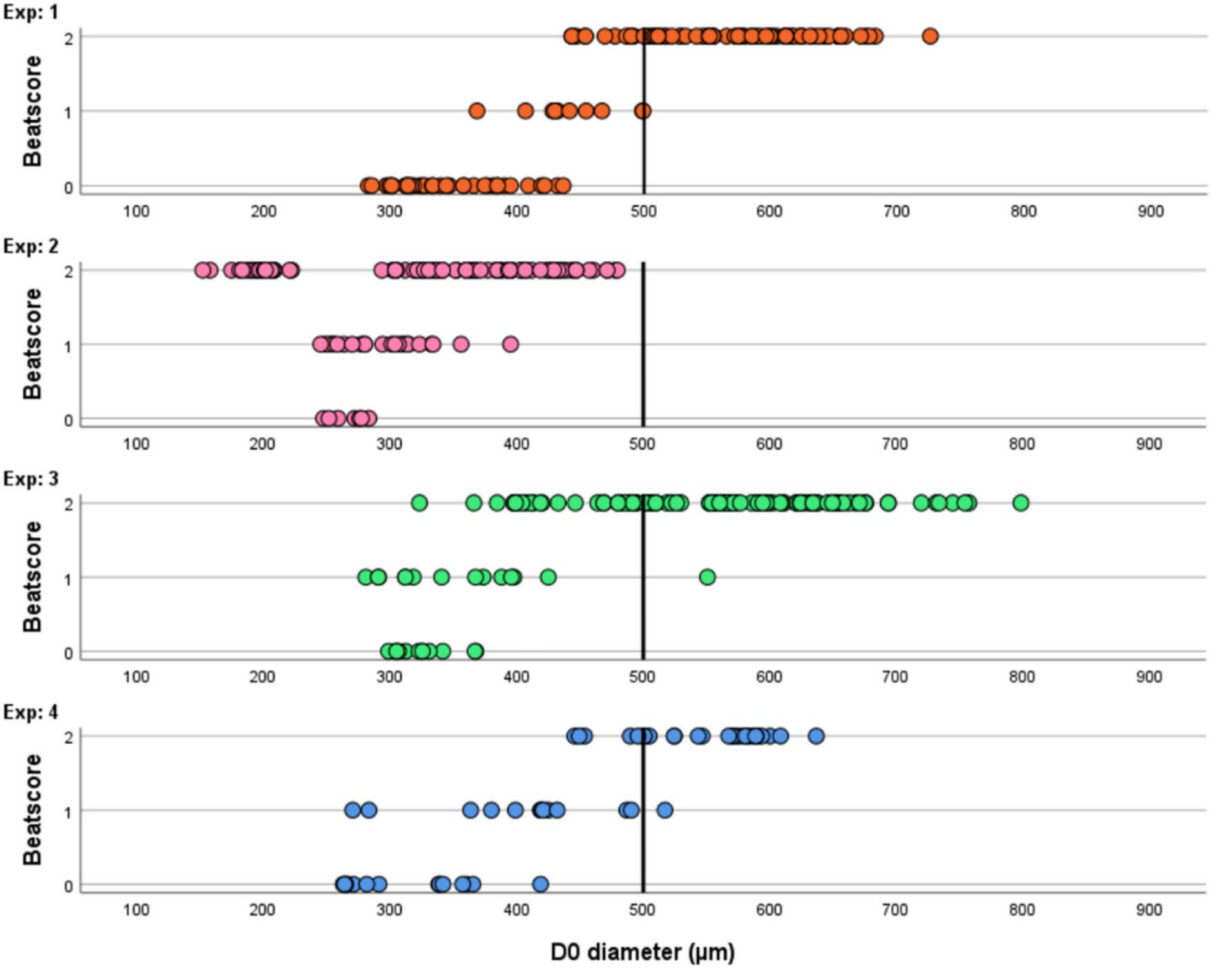
Beating of 3D cardiomyocyte spheroids on Day 7 (D7) of the PluriLum assay represented as a function of the diameter of the embryoid bodies (EBs) at Day 0 (D0) of each individual experiment. Results of four individual experiments are shown and each dot represents one individual EB. The values of the y-axis represent the beat score values: (0) meaning “No Beat” on D7; (1) meaning “Partial Beat” on D7; or (2) meaning “Full Beat” at D7. The x-axis represents the diameter (µm) of the individual EBs on D0. Vertical black lines at the value of 500 µm are placed on the x-axis of all experiments to indicate lower threshold for differentiation success

**Fig. 2:**
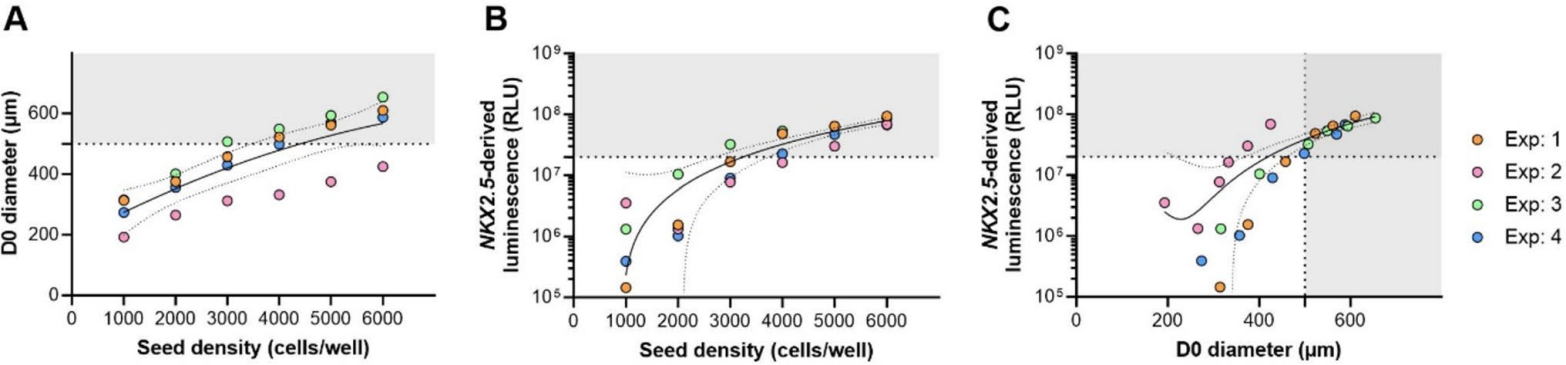
Data from four individual experiments that describe the relationship between **A** average embryoid bodies (EBs) diameter at Day 0 (D0) and seeding density cells/well on Day-1 (D-1), **B** *NKX2.5-*derived relative luminescence units (RLU) on Day 7 (D7) of the differentiation protocol and the initial average seed density, and **C** *NKX2.5-*derived luminescence on D7 and D0 average diameter. Results are fitted to a quadratic regression with fit and 95% confidence interval bands plotted in each graph. Lower limits for D0 diameter (500 µm) and *NKX2.5*-derived luminescence (2 × 10^7^ RLU) are represented as horizontal/vertical dotted lines

For experiments related to the effects of EB size on differentiation efficiency (Figs. 1 and 2), hiPSCs were seeded at different cell densities (1000, 2000, 3000, 4000, 5000 or 6000 cells/well) on D-1 to generate EBs of varying size.

The seeded plates were then incubated overnight at 37 °C and 5% CO_2_, and after a 19h (± 2h) overnight incubation (Day 0, D0), EBs had formed in the bottom of the conical wells, and the medium was exchanged into D0 differentiation medium.

After 24 h of incubation (Day 1, D1), medium was changed to TS medium. After an additional 24 h incubation (Day 2, D2), medium was changed into WNT-containing TS medium. After 24 h (Day 3, D3), the medium was changed again to TS medium and left to incubate for extra 72 h until Day 6 (D6). At D6, medium was replaced with fresh TS medium. The differentiation was assessed after an additional 24 h, i.e., on Day 7 (D7), where the experiment was terminated.

The original assay protocol (Lauschke et al. 2021b) runs for a total of 8 days (D-1 to D7). To assess the sensitivity of a shortened protocol, a set of experiments were conducted shortening the original protocol by 24 h, thus terminating on D6.

### Chemical exposures

Epoxiconazole, a*t*RA, and 13-*cis*RA were prepared in a stock solution in DMSO concentrated by a factor of 1000 relative to the highest final exposure concentration (40 mM epoxiconazole, 1 mM a*t*RA and 3 mM 13-*cis*RA). For exposure experiments, dilutions in DMSO were added in a ratio of 1:1000 to the respective medium on the experimental days D1, D2 and D3, as well as on D6 for experiments terminating on D7, allowing for a constant vehicle (V/V) concentration of 0.1% DMSO in all wells. For each individual experimental condition, 6 EBs were exposed.

### Imaging and sizing of embryoid bodies

The imaging of the individual EBs was performed on D0, on a BioTek Cytation 5 Cell Imaging Multimode Reader (Agilent Technologies, Santa Clara, USA), using a 4X objective. Size measurement was done using BioTek Gen5 Image Prime Software, by masking the edge of the EBs and calculating the diameter. Subsequent analog measurements were made to verify the quality of the size measurements using ImageJ (NIH, USA).

### Scoring of embryoid body contraction

Upon completion of the assay, the contractility of each individual EB was assessed by visually evaluating the contractility using a light microscope and a 4X objective (Nikon Eclipse Ts2, Tokyo, Japan). Each EB was observed for maximum 15 s, or until beating was established.

The contractility was scored using the criteria: “Full Beat” (whole EB visibly contracting), “Partial Beat” (if contraction was less than whole), or “No Beat” (no visible contraction).

### Analysis of *NKX2.5* activation by luminescence measurements

Quantification of *NKX2.5* activation in the individual EBs was analyzed by luminescence measurements using the Promega Nano-Glo^®^ Luciferase Assay System (Promega, Wisconsin, USA). The EBs were washed by removing 50 µL medium from each well and adding 150 µL DPBS. The EBs were transferred in a volume of 40 µL into flat bottomed white 96-well plates for luminescence measurements. The EBs were incubated with 40 µL papain (Merck, Darmstadt, Germany) at a final activity of 40 U/mL for at least 1.5h. After incubation, the EBs were dissociated into a cell suspension by pipetting up and down 20 times (Fischer et al. 2018). After dissociation, 40 µL cell suspension was transferred into a flat bottomed white 96-well plates containing 40 µL of Nano-Glo^®^ Luciferase Assay Substrate followed by pipetting up and down. To the remaining 40 µL of cell suspensions, 40 µL of CellTiter-Glo^®^ (Promega) were added and plates were shaken on an orbital shaker for 10 min before stabilizing for 20 min. Measurements of both Nano-Glo^®^ and CellTiter-Glo^®^ luminescence were performed on a PerkinElmer EnSpire 2300 Multimode Microplate Reader (PerkinElmer, Inc., Massachusetts, USA).

### Data processing and statistical analysis

Statistical analysis on luminescence data from chemical exposures was performed using GraphPad Prism 10 (version 10.0.3). All experiments were performed in three independent experiments using different passages of cells. Each experimental condition contained six technical replicates (EBs) in each experiment.

The value for each experimental condition is the average of the normalized relative luminescence units (RLU) of the technical replicates. Results are presented as mean ± standard deviation.

Statistical analysis was performed using two-way ANOVA, followed by multiple comparisons using the Bonferroni post hoc test.

## Results

### Impact of diameter at Day 0 on success of cardiomyocyte differentiation

We investigated the relationship between the EBs diameter measured at D0 and the cardiac spheroid contractility on D7. Data of the four independent experiments are shown in Fig. 1, with each dot representing the diameter of an EB on D0 and its corresponding beat score on D7 post-differentiation.

Experiments #1, 3, and 4 showed similar tendencies in the measured outcomes of spheroid contractility (Fig. 1). A threshold of 500 µm, represented by the vertical black line in the graphs of each experiment in Fig. 1, was defined as the lower limit for an adequate differentiation, as all EBs with a D0 diameter above this limit yielded (fully or partially) contracting spheres on D7. Out of these, only 3 EBs with a D0 diameter above 500 µm on D0, equivalent to 2.3%, resulted in a partial beating on D7 and the remaining EBs were fully beating.

Experiment #2 (pink dots) displayed smaller D0 diameters in all seeded conditions, with no EBs exceeding the set threshold of 500 µm. A dissimilar pattern between size and cardiac differentiation success was also noted in this experiment, with very small EBs (diameter < 250 µM at D0) differentiating into beating cardiomyocytes.

### Relationship between seeding density, D0 diameter, and cardiomyocyte differentiation

We further investigated the relationship between the seeding density on D-1, the diameter of the EBs on D0, and the resulting *NKX2.5-*derived luminescence read-out on D7 as a measure of cardiac differentiation success of the same four experiments (Fig. 2).

As expected, we observed increasing average EB diameters on D0 when seeding density increased from 1000 to 6000 cells/well for all experiments (Fig. 2A). This increase was close to linear.

We demonstrated that seeding higher cell densities resulted in increased luminescence output of the spheroids on D7 (Fig. 2B). This increase in luminescence deviated more from linearity at lower seeding densities, with a large variation between experiments that leveled off above a seeding density of 4000 cells/well. The decline in variation of luminescence output with the increase in seed density is denoted by the plotted 95% confidence interval band of the fitted data from all four experiments in Fig. 2B. An adequate variation of luminescence levels is observed above 2 × 10^7^ RLU (horizontal dotted line on graphs from Fig. 2B and C). As seen in Fig. 2C, when applying both D0 diameter (500 µm) and *NKX2.5*-derived luminescence (2 × 10^7^ RLU) thresholds, we demonstrate that an increase in D0 diameter, dependent on cell seeding at D-1, translated linearly to an increase of RLU at D7 for EBs above 500 µm. Below diameters of 500 µm, the variance of luminescent output was much higher.

Of note, experiment #2 (pink dots), which did not yield any EBs with a D0 diameter above 500 µm at any seeding densities (Figs. 1 and 2A), also diverged the most from the remaining three experiments in terms of *NKX2.5*-derived luminescence at D7 (Fig. 2B and C), illustrating an experiment that would not pass the quality control check set by the defined lower limit of D0 diameter, and would, thus, be disqualified for experimental studies already from D0.

### Relationship between initial cell density and effect reproducibility

Next, we investigated the effects of initial seed densities on the concentration–response curves of the fungicide epoxiconazole in the PluriLum assay. We did this by seeding increasing cell density on D1 (3000, 4000, 5000, and 6000 cells/well), leading to the formation of EBs of different size, and then exposing these to increasing concentrations of epoxiconazole.

**Fig. 3:**
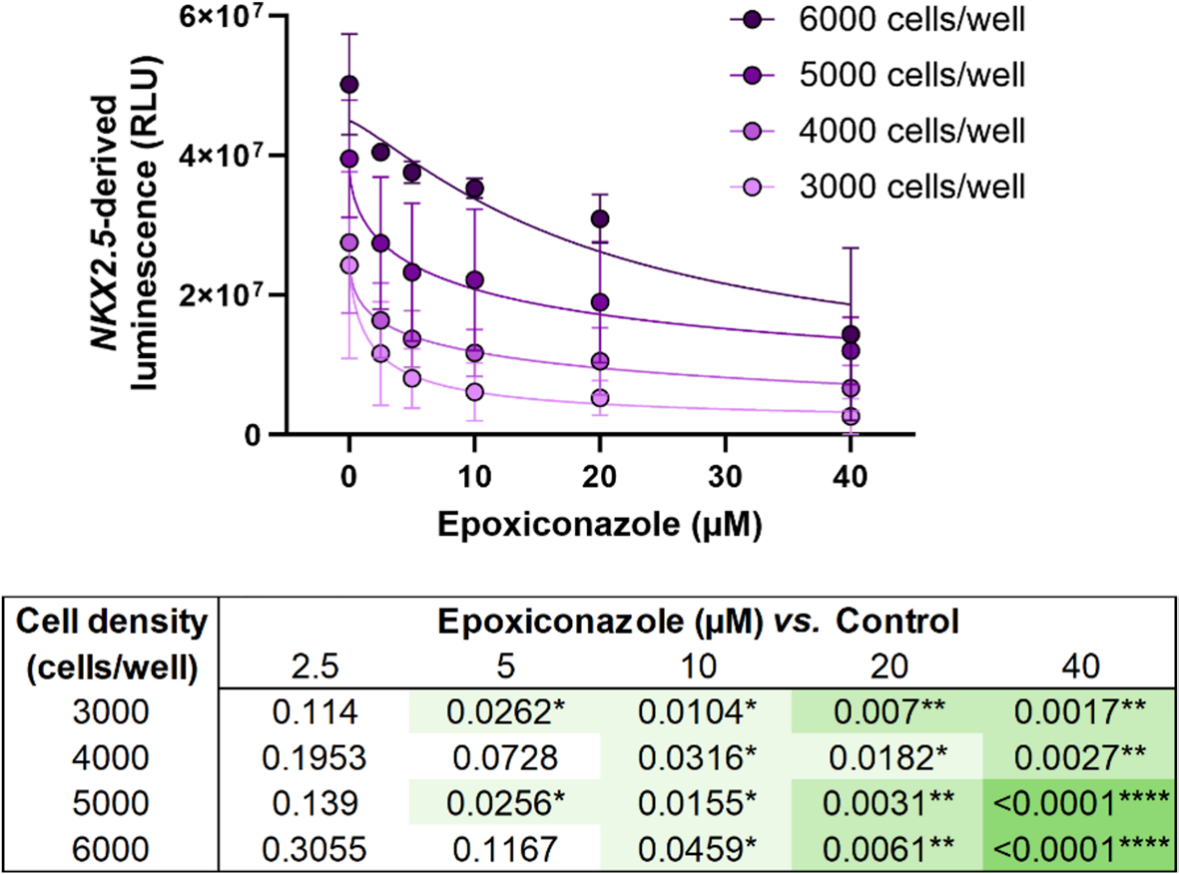
Dose–response curves showing effects on *NKX2.5-*derived luminescence caused by exposure to increasing concentrations of epoxiconazole, at different initial cell densities (3000, 4000, 5000, and 6000 cells/well). Table shows adjusted *p* values for each exposure concentration of epoxiconazole at different initial seeding densities. *p < 0.05, **p < 0.01, ***p < 0.001, ****p < 0.0001 *vs.* control

As expected, we observed that higher cell densities at the start of the experiments resulted in increasing average baseline luminescence output levels at D7, thus establishing different starting points for the concentration–response curves (Fig. 3).

Statistically significant effects were determined for the tested exposure range for all initial cell densities (Fig. 3). However, the slopes of the individual fitted concentration–response curves obtained at initial seeding of 6000 cells/well were less pronounced that for the lower densities, i.e., 3000, 4000, and 5000 cells/well. All seeding densities resulted in statistically significant effects following exposure to 10 µM epoxiconazole and higher concentrations. The response was also found to be significant already at 5 µM epoxiconazole for experiments with initial cell densities of 3000 and 5000 cells/well. The most significant effects were noted at 20 and 40 µM epoxiconazole at the higher seeding densities of 5000 and 6000 cells/well (lowest *p* values).

### Shortening of the plurilum protocol

**Fig. 4:**
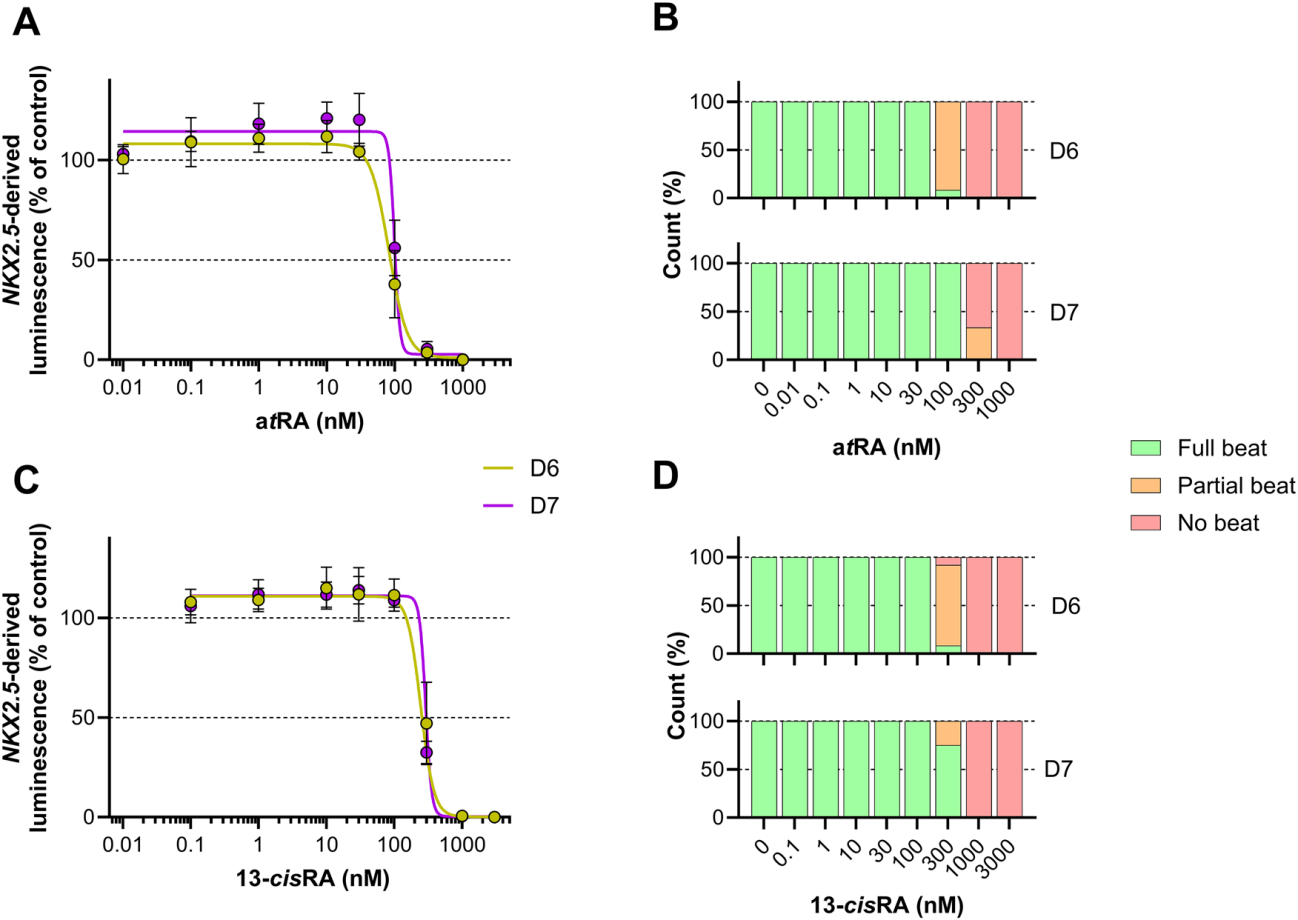
Comparison of effects of exposure to all-*trans* (a*t*RA) and 13-*cis* retinoic acid (13-*cis*RA) in the PluriLum assay upon assay completion on D6 and D7. **A** The effects of exposure to a*t*RA on average *NKX2.5-*derived luminescence. **B** Visualization of contractility as an effect of exposure to a*t*RA. **C** The effects of exposure to 13-*cis*RA on average *NKX2.5-*derived luminescence. **D** Visualization of contractility as an effect of exposure to 13-*cis*RA

We investigated the impact of reducing the length of the PluriLum assay protocol by 24 h, ending the experiment on D6 instead of D7 of the differentiation protocol. To do this, we compared the effects of exposure to increasing concentrations of a*t*RA (0.01–1000 nM) and 13-*cis*RA (0.01–3000 nM) measured at D6 and D7, on both *NKX2.5-*derived luminescence (Fig. 4A and C) and beating of the cardio spheres (Fig. 4B and D).

When comparing the luminescence read-out measured at D6 and D7, similar patterns of effect were observed for both a*t*RA and 13-*cis*RA. The inhibitory concentration of 20% (IC_20_) of a*t*RA was lower at D6 (59 nM) when compared to D7 (92 nM; Fig. 4A). Likewise, exposure to 13-*cis*RA showed an IC_20_ of 199 nM and 264 nM on D6 and D7, respectively (Fig. 4C). These results indicate that the sensitivity of the assay is not negatively affected, but rather increased on D6 compared to D7 under these experimental conditions.

In line with this, an effect was picked up by the beat score at 100 nM a*t*RA on D6 and only at 300 nM at D7 (Fig. 4B). For 13-*cis*RA beating outcomes started to be affected at the same concentration level (300 nM) on both days, but with a larger proportion of spheroids affected on D6 (92%) compared to D7 (25%) partially or no beating cardio spheres, respectively; Fig. 4D.

### Dissociation of cardio spheres for simultaneous assessment of multiple endpoints

We have developed a protocol for dissociating the cardio spheres into a single cell suspension to be able to split the individual spheroids into various samples in a uniform manner. This enables us to assess multiple endpoints using the same sphere, besides the quantification of the *NKX2.5-*derived luminescence.

**Fig. 5:**
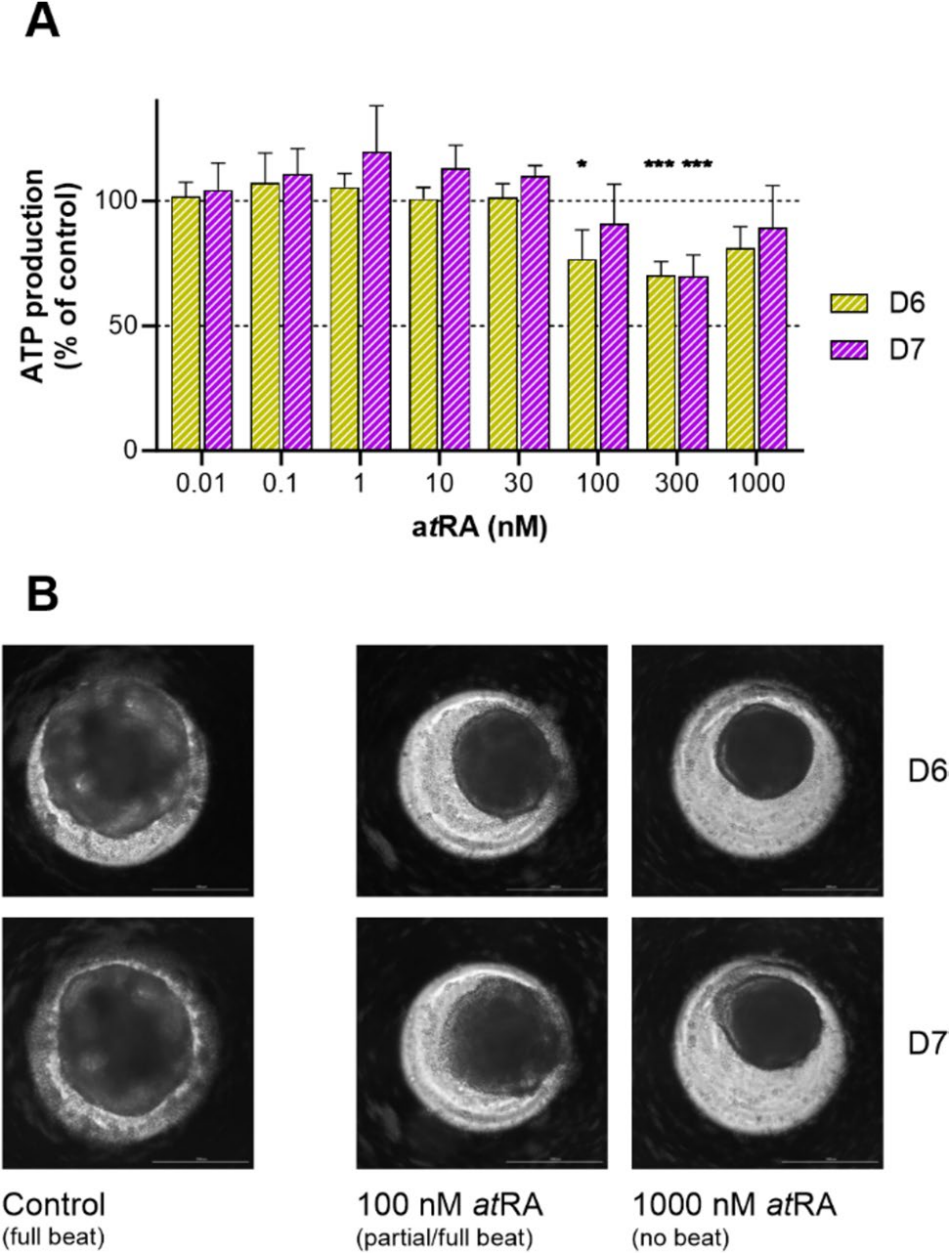
Comparison of effects from exposure to increasing concentrations of a*t*RA in the PluriLum assay, with comparison on assay completion on Days 6 and 7 (D6 and D7). **A** Effects of exposure to a*t*RA on average ATP levels (normalized to control) of dissociated spheres. **B** Representative images of cardio spheres in control conditions and exposed spheres to 100 and 1000 nM atRA (4X magnification)

As proof of concept, we measured ATP levels in dissociated samples after exposure to a*t*RA at increasing concentrations (Fig. 5), using the same experimental plates exposed for assessment of *NKX2.5*-derived luminescence and beat score (Fig. 4A and B). We found that exposure to a*t*RA led to a significant decline in ATP levels from 100 nM measured at D6 and 300 nM measured at D7, but not at the highest tested concentration of 1000 nM (Fig. 5A). A visual confirmation of the spheroids morphology after exposure to these levels of a*t*RA is represented in Fig. 5B. The morphology of spheres on D6 and D7 does not appear to differ greatly, but a decrease in size is evident both at 100 and 1000 nM a*t*RA compared to control. Of note, spheroids show no contractility and display a more compacted and smoother morphology at 1000 nM a*t*RA.

## Discussion

When utilizing stem cells undergoing differentiation as the foundation of any assay, rigorous protocols and quality checks are key to ensure reproducible and biologically relevant results (Kugler et al. 2017; Velasco et al. 2020; Li et al. 2022). The desired differentiation of an EB toward a cardio sphere in the PluriLum assay is affected by several factors preceding assay initiation—cell morphology, confluency, passage number, and general handling of the stem cells that all must lie within defined standards in order for the assay to run successfully. However, even with standardized procedures in setting up the assay, variation between the final cardio spheres is inevitable, creating intra-experimental variation, as well as variation between experimental setups.

Effects of aggregated EB sizes on 3D cardiomyocyte differentiation are not a new phenomenon—several groups have described how aggregate diameter plays a significant role in the success of cardiac differentiation (Branco et al. 2020). The cellular processes involved in 3D cardiac differentiation of hPSCs have been shown to harbor cell line and protocol specific differences, that makes direct comparison of levels of factors and other growth conditions between uses difficult.

In this work, we have substantiated and implemented an in-assay quality standard of minimum EB diameter of 500 µm on D0 of the protocol to enhance consistency in the anticipated performance of an experimental setup prior to chemical exposure. We show that, when applying this lower limit, we can expect, with high confidence, a uniform and successful differentiation into a contracting cardio sphere on D7 (Fig. 1). Although smaller sized EBs may also successfully differentiate, they do so with lower fidelity on average. We observe that EBs emit *NKX2.5-*derived luminescence in a close to linear fashion on D7 for EBs with a D0 diameter above 500 µm (Fig. 2C). We find that EBs with a D0 diameter below this 500 µm threshold are associated with higher variability in luminescent output, which we interpret as stemming from increased variation in cardiac differentiation success at smaller EB sizes. Both the successful cardiomyocyte differentiation as well as consistency of the luminescent output are the foundation of the PluriLum assay. By introducing this quality standard related to key features of the PluriLum assay, it becomes more robust and better suited for future toxicity applications. Furthermore, it allows for the resection of experiments with high probability of failure already at D0 of the protocol, reducing the costs by avoiding full runs of substandard experiments.

Other groups have reported that EB sizes affect spontaneous lineage specification, where smaller aggregate diameters tend to differentiate toward the ectoderm and endoderm lineages whereas larger EBs tend to differentiate toward the mesoderm lineage (Brickman and Serup 2017; Zeevaert et al. 2020). This could potentially play a role in the baseline ability of EBs in the PluriLum assay to successfully develop into cardiomyocytes at different EB sizes and to the lower differentiation success at sub-500 µm diameters.

Similar findings to ours have been reported in cases where cardiac 3D constructs form with less efficiency bellow 600 µm (200 µm, 400 µm), while larger spheroids have a higher likelihood of successful differentiation (600 µm, 800 µm, 1000 µm) (Hoang et al. 2021). In contrast, others have reported that cardiac differentiation performed best at lower EB diameters of 250–350 µm, where larger EBs perform sub-optimally (Mohr et al. 2010).

The case-specific nature of EBs differentiation capacity makes it difficult to draw direct parallels between cell lines and protocols used by other groups.

The PluriLum assay protocol is under normal experimental conditions set up with a density of 5000 cells/well. To demonstrate how EB size plays an important role in chemical exposures responses, we compared the outcome of the PluriLum assay following different initial cell densities (from 3000 to 6000 cells/well), yielding variable sizes of EBs for an exposure study. The fungicide epoxiconazole was used for this comparison, as it has previously been shown to provoke a potent response in the PluriLum assay (Lauschke et al. 2021a; Treschow et al. 2024).

We found that epoxiconazole acted in a concentration-dependent manner for all seed densities (Fig. 3) with statistically significant effects at 5 µM for the initial seeding densities of 3000 and 5000 cells/well. While this can be interpreted as a similar display of sensitivity, it might be relevant to highlight that effects at smaller EBs sizes can be confounded with poor cardiac differentiation, and thus it cannot be excluded that the observed significance at 3000 cells/well can be a result of that. When further looking at the results, we also observed that seed densities of 5000 and 6000 cells/well yielded higher certainty in exposure effects (i.e., lower *p* values) at 40 µM epoxiconazole exposure when compared to the lower seeding conditions. Nevertheless, the less pronounced slope of the concentration–response curve and the lack of effect of exposure to 5 µM epoxiconazole in the experiments seeded with 6000 cells/well could be due to decreasing sensitivity at larger EBs sizes. This means that, although setting a lower limit for EBs size at D0 may be beneficial for quality of cardiomyocyte differentiation, larger EBs might also lead to decreased ability of the assay to pick up effects of chemical exposure. This lower sensitivity in EBs with bigger dimensions could stem from the inability of the test compounds to diffuse through larger and/or more compact 3D structures, as suggested by others (Kim et al. 2013; Nath et al. 2017). Our findings suggest that an investigation into reduced sensitivity to certain chemicals at larger EB sizes would be relevant in the future.

Taken together, EBs seeded with an initial density of 5000 cells/well were demonstrated to display simultaneously good sensitivity and higher certainty of exposure effects.

The known teratogen a*t*RA has previously been shown to trigger a response in the PluriLum assay (Treschow et al. 2024). In addition, the isomer 13-*cis*RA is also known to have teratogenic effects and is known to provoke responses in assays comparable to the PluriLum assay (Liu et al. 2018). Here we used both isomers to compare the performance of the PluriLum assay upon termination of the differentiation protocol on D6 and D7. We showed that the exposure to a*t*RA and 13-*cis*RA provoked a clearer response on D6 compared to D7 with regards to cardio sphere contractility, as well as a slightly more sensitive response for *NKX2.5-*related luminescence (Fig. 4). This suggests that the assay displays increased sensitivity upon termination on D6 compared to D7.

Work performed during the development of the PluriLum assay protocol showed that the expression levels of *NKX2.5* peaks on Day D6 and D7 of the assay protocol (Lauschke et al. 2020; Treschow et al. 2024), and that the expression is at a comparable level. When exposing EBs to various chemicals during differentiation, one consequence of the exposure could be a delay of the cardiac differentiation, which would shift the expression of cardiac specific genes toward a later timepoint. This delay of cardiac differentiation and onset of beating has been previously used as an endpoint for assessing developmental toxicity by Palpant et al. (2015).

In the PluriLum assay, a chemically induced delay in expression from D7 levels to D6 levels would result in a comparable level of *NKX2.5* oppression, while a delay from D6 levels toward D5 levels would yield a relatively larger difference in total expression. By changing the length of the protocol by 24 h, we, thus, decrease the risk of missing effects due to chemical exposures and reporting false negative results, while also reducing the time and costs associated with running the assay. The reduction in protocol length further emphasizes the difference to comparable assays, which typically has a protocol length of 10–14 day (Treschow et al. 2024).

To maximize the output of the PluriLum assay, we have introduced an enzymatic dissociation step using papain that facilitates the splitting of the individual spheroids into several equal parts. This addition makes measurements of endpoints other than *NKX2.5-derived* luminescence possible on a single well, here exemplified by including measurements of ATP levels upon exposure to a*t*RA. The possibility of including more than one endpoint makes the assay more versatile and increases the value of each single experimental run.

We showed that the trend of ATP levels on D6 and D7 is the same, but levels are significantly reduced by exposure to 100 and 300 nM a*t*RA on D6 and only at 300 nM on D7, further substantiating the higher sensitivity of D6 termination. On the other hand, exposure to 1000 nM a*t*RA resulted in non-significant reductions on both D6 and D7.

Disruption of mesoderm differentiation due to a*t*RA exposure has been previously reported, as it has been shown to alternate differentiation pathways toward an endoderm lineage (Hoang et al. 2021). We observed that the morphology of the spheroid at 100 nM and 1000 nM showed clear differences in density and shape. The increasing concentrations of a*t*RA led to a decline in ATP levels but only up to 300 nM, but did not facilitate a dose-dependent response, as it recovered at the highest concentration of 1000 nM. This indicates that the observed reduction is most likely not due to loss of viable cells. Instead, this U-shaped trend may be a result of a lower energy demand by the hampered cardiomyocyte differentiation, followed by a change of the differentiation fate of the stem cells. In other words, the rebound in ATP levels at 1000 nM exposure could stem from a full commitment to an alternate differentiation pathway compared to intermediate exposure concentrations of 100 nM and 300 nM. Nevertheless, further studies are needed to determine the differential fate of EBs exposed to a*t*RA and other potential teratogens with similar modes of action.

## Conclusion

With this work, we have increased the robustness, sensitivity, and speed of the PluriLum assay, by shortening the assay protocol and adding a minimum acceptable EB size threshold at the beginning of the experiments. We have also established a method of enzymatic dissociation that enables the assessment of multiple endpoints in a single EB, maximizing the utilization of each individual experiment.

By introducing standardization and optimization steps to the protocol, we worked toward improving the predictive and high-throughput potential of the PluriLum assay, aiming at becoming a generally accepted NAM, suitable for the screening of embryonic and developmental toxicants.

## Data Availability

The datasets generated during and/or analyzed during the current study are available from the corresponding author upon reasonable request.
